# Psychophysiological factors as predictors of second language writing achievement in a computer-based test

**DOI:** 10.3389/fpsyg.2022.958938

**Published:** 2022-10-19

**Authors:** Shu-Ping Lee, Shujen Lee Chang, Hui-Kai Su, Zhen-Yang Cui, Shin-Da Lee

**Affiliations:** ^1^Department of Foreign Language, Asia University, Taichung, Taiwan; ^2^Department of Psychology, Asia University, Taichung, Taiwan; ^3^Department of Electrical Engineering, National Formosa University, Yunlin, Taiwan; ^4^School of Rehabilitation Science, Weifang Medical University, Shandong, China; ^5^Department of Physical Therapy, Graduate Institute of Rehabilitation Science, China Medical University, Taichung, Taiwan; ^6^Department of Physical Therapy, Asia University, Taichung, Taiwan

**Keywords:** anxiety, cognitive load, second language writing, sleep, personality

## Abstract

Sleep quality, personality, and cognitive load potentially increase second language writing (SLW) anxiety and subsequently affect SLW achievement. This study investigates the predictions of sleep quality, personality (social inhibition/ negative affectivity), and cognitive load (content/ computer) toward SLW anxiety and achievement in a computer-based test. Participants included 172 voluntary undergraduates majoring in English as foreign language. SLW anxiety in a computer-based test, sleep disturbance, personality and cognitive load was assessed with the SLW Anxiety Inventory, Pittsburg Sleep Quality Index, Type-D Personality, and cognitive load questionnaires. A structural equation modeling approach was applied to examine the interdependence among the observed variables. An adequate-fit SLW anxiety model was built (*X^2^* = 6.37, *df* = 6, *p* = 0.383, *NFI* = 0.97, *CFI* = 1.00, *RMSEA* = 0.02; R-squared multiple correlations: SLW anxiety in a computer-based test = 0.19, computer-based SLW achievement = 0.07). The structural model showed that sleep disturbance (+0.17), social inhibition personality (+0.31), and computer-induced cognitive load (+0.16) were significant predictors of SLW anxiety in a computer-based test. Subsequently, SLW anxiety in a computer-based test (−0.16) and computer-induced cognitive load (−0.16) were significant negative predictors of computer-based SLW achievement.

## Introduction

Second language anxiety is responsible for uncomfortable learning experiences (Horwitz et al., 1986) and plays a critical role in second language learning. Second language anxiety substantially affects second language achievement, including speaking, listening, reading, and writing (Horwitz, 1986; Gregersen, 2005; Li and Wei, 2022). Second language writing (SLW) anxiety is related to second language writing performance (Gungle and Taylor, 1989; Cheng, 2004). Second language writing anxiety is a strong negative predictor of second language writing achievement (Lee and Krashen, 1997; Woodrow, 2011; Park and French, 2013).

Computer-based writing tests, computer-based online learning or computer-based feedback were frequently applied and studied (Lee et al., 2012, 2015; Lu, 2022; Sherafati and Mahmoudi Largani, 2022; Zhao, 2022). The foreign language writing test anxiety was further induced by the computer-based testing system of a foreign language (Stricker et al., 2004). In a computer-based test, second language writing anxiety also involves computer anxiety, a situation-specific state of anxiety occurring when people are frightened of using computers or even just considering using computers (Simonson et al., 1987). It is still unknown whether second language writing anxiety in a computer-based test would be negatively associated with computer-based second language writing achievement.

Poor sleep quality is reported to be correlated with poor academic performance at various education levels including middle school, high school, and college (Trockel et al., 2000; Gaultney, 2010; Pagel and Kwiatkowski, 2010). Sleep disorders were reported as a risk to language learning and performance from a scoping reviewing article (McGregor and Alper, 2015). Sleep and anxiety as a reciprocal process influence one another and collectively directly impair academic performance of English as a second language (Hamilton et al., 2021). The percentage of college students with self-reported sleep problems rose from 24% in 1978 to 53% in 1988 (Hicks et al., 1989), to 44% in 2003 (Yang, 2003), and to 60% in 2010 (Lund et al., 2010). It is still unknown whether a greater level of sleep disturbance in college students may be associated with higher second language writing test anxiety and lower second language writing achievement in a computer-based test.

Personal characteristics and personalities play a crucial role in the second language writing process (Eom and Papi, 2022). Social inhibition, also known as behavioral inhibition, is the tendency to avoid certain behaviors in social situations, including social interactions. Social inhibition personality refers to a strong inclination to avoid the expression of emotions or interactions in social situations to prevent others’ disapproval; people having a strong social inhibition personality tend to feel stressed and insecure when interacting with others (Gest, 1997). It has been shown that social inhibition personality negatively impacts communicative foreign language performance (Patterson and Ritts, 1997; King and Smith, 2017), but it is still unknown whether social inhibition personality negatively impacts second language writing anxiety and/or achievement.

Negative affectivity personality refers to the tendency of experiencing negative emotions most of the time; people having a strong negative affectivity personality tend to have a negative view of self and the world around (Watson and Pennebaker, 1989; Denollet, 1991). Negative affectivity is thought to be a vulnerability factor for depressive and anxiety symptoms and may be more related to anxiety rather than depression (Iqbal and Dar, 2015). An empirical evidence suggested that there is a clinically-relevant interplay between negative affectivity and anxiety sensitivity (Zvolensky et al., 2016). It is unknown whether people having a stronger negative affectivity personality may have higher SLW anxiety and achievements.

Cognitive load refers to the cognitive resources used in the executive control of working memory in learning situations and is defined as the allocation of working memory resources to organize content elements and conduct content element interactivity in a learning situation (Paas et al., 2004; Sweller, 2010). Learning to write in a foreign language is a complex cognitive process and the quality of SLW can be improved by optimizing the cognitive (working memory) load (Jiang and Kalyuga, 2022). Computer-based online language learning increases learners’ cognitive load and negatively impacts learners’ foreign language anxiety (Kock, 2011; Zhao, 2022). Taking a computer-based test of SLW is relatively more complex, since students are required to efficiently manage cognitive resources for both writing content and computer operating. The content (intrinsic) and computer (extraneous) cognitive load related to SLW in a computer-based test can theoretically be higher for students facing computer-based learning or e-learning (Hollender et al., 2010). Writing demands excessive cognitive resources since it is a high-level conceptual process for most writers and involves the complicated steps of planning, revising, and deciding about content selection as well as organization (Kellogg, 2008). It is unclear whether people having a higher content (intrinsic) or computer (extraneous) cognitive load may have higher SLW anxiety and achievements.

The second language writing process is an extremely complex task with various psychophysiological or cognitive challenges (Bruning and Horn, 2000; Schweiker-Marra and Marra, 2000). Thus, this study examines psychophysiological factors as predictors of sleep disturbance, personality (social inhibition and negative affectivity), and cognitive load (content and computer) on SLW anxiety and achievement in a computer-based test through single variate correlation and multiple variate structural equation model (SEM) analyzes. Thus, we hypothesized that more sleep disturbance, social inhibition and negative affectivity personalities, and higher content and computer cognitive load may be associated with higher SLW anxiety and lower second language writing achievement in a computer-based test.

## Materials and methods

### Participants

Participants were 172 voluntary undergraduates with English major (45 males, 127 females, 90 freshmen, 82 sophomores, average age 19.26, *SD* = 1.28), who enrolled in multiple sections of a required course titled, Introduction to English Writing, in the Department of Foreign Languages and Literature of a university in central Taiwan. Participants, whose native language is Chinese, have learned English as a foreign language from middle and high schools for at least 6 years. Before the study began, the study was reviewed and approved by Asia University Medical Research Ethics Committee. Written Informed Consent Forms reviewed by Asia University Medical Research Ethics Committee were obtained from the all participants in classroom.

### Variables and measurements

All variables in this study were measured by self-reported surveys, except that computer-based SLW achievement was measured through a computer-based test. Surveys were translated from English to Chinese with back-translation methods and presented to participants in Chinese and English at the same time.

#### Second language writing anxiety in a computer-based test

Second language writing (SLW) anxiety in a computer-based test refers to the anxiety caused by writing in English as a second language in a computer-based test situation. SLW anxiety in a computer-based test was measured by the SLW Anxiety Inventory (range 22–110) with 22 items on a 5-point scale, with higher scores indicating higher anxiety (Cheng, 2004). Example items include “I feel my heart pounding when I write English compositions under time constraints,” and “While writing English compositions, I feel worried and uneasy if I know they will be evaluated.” This scale has adequate validity and high reliability. It is highly correlated with the most commonly used measurement instruments of L2 writing anxiety scales, such as the L2 version of the Daly–Miller Writing Apprehension Test (*r* = 0.79) and Writer’s Block Questionnaire (*r* = 0.69; Daly and Miller, 1975; Rose, 1984; Cheng, 2004). The SLW Anxiety Inventory scale has high test–retest reliability (*r* = 0.85) and internal consistency (Cronbach’s *α* = 0.91; *N* = 421). In this study, the internal consistency of SLW Anxiety Inventory was also high (Cronbach’s *α* = 0.95; *N* = 172).

#### Computer-based second language writing achievement

The second language writing (SLW) achievement in a computer-based test is the score a participant earned from the SLW examination through a computer-based test, a required test for the course in which participants enrolled. The scores were the average scores rated by two professors and one graduate student. The examination included three similar writing questions selected from TOFEL. These questions were classified as easy and appropriate for the participants in this study. The score of SLW achievement in a computer-based test (range 0–15) was the sum of three writing questions (score 0–5 each). The inter-rater reliabilities of SLW achievement scores among the three raters were significantly high (*r* = 0.82, 0.75, 0.74; *p* < 0.001 for all correlations).

#### Sleep disturbance

Sleep disturbance refers to participants’ experiences of sleeping troubles during the last month of survey and was measured by the sleep disturbance subscale of the Pittsburgh Sleep Quality Index (PSQI); (Buysse et al., 1988). The sleep disturbance subscale asks participants how often, and why, they had trouble sleeping. Example items were “Wake up in the middle of the night or early morning” or “Had bad dreams” with choices of “not during the past month” (score 0), “less than once a week” (score 1), “once or twice a week” (score 2), and “three or more times a week” (score 3). The sum of nine-item scores was calculated and sleep disturbance was recorded as 0 (sum score 0), 1 (sum score 1–9), 2 (sum score 10–18), and 3 (sum score 19–27), with the higher scores indicating having a higher level of sleep disturbance. The validity of PSQI was adequate as the global PSQI scores differed significantly between the healthy group and patient groups with depression or disorders of excessive somnolence diseases; the test–retest reliability of PSQI was sufficiently stable (Buysse et al., 1988).

#### Social inhibition and negative affectivity personality

The Type-D personality scale assesses the traits of social inhibition and negative affectivity personality (Sweller, 2010). The social inhibition subscale evaluates discomfort in social interactions and lack of social poise, while the negative affectivity subscale measures dysphoria, worry, and irritability. Example items of the social inhibition subscale include “I often feel inhibited in social interactions” and “I find it hard to start a conversation.” Example items of negative affectivity subscale include “I often make a fuss about unimportant things” and “I often feel unhappy.” Each subscale (range 0–28) includes seven 5-point-items. A higher total subscale score indicates a stronger personality trait in the respective dimension.

The Type-D personality scale is equally applicable to Chinese and various ethnic groups, although originally developed with Belgian patients (Pedersen and Denollet, 2004; Yu et al., 2010). The construct validity of the TDP scale was confirmed with the NEO Five-Factor Inventory and Eysenck Personality Questionnaire (Costa and McCrae, 1992). Social inhibition and negative affectivity personality subscales were internally consistent (Cronbach’s *α* = 0.86 and 0.88; *N* = 3,678) with a stable test–retest reliability (*r* = 0.82 and 0.72). In this study, the internal consistencies of social inhibition and negative affectivity personality were also high (Cronbach’s *α* = 0.98 and 0.79; *N* = 172).

#### Content and computer cognitive load

Cognitive load refers to the allocation of cognitive resources or mental effort for to-be-learned content and element interactivity (Paas et al., 2004; Sweller, 2010). In this study, we examined cognitive load concerning two aspects: the writing content and test method (a computer-based test). Each aspect of cognitive load was measured by a widely used self-report cognitive load measure adapted from previous studies (Brunken et al., 2003; Deleeuw and Mayer, 2008). E. The content cognitive load asked participants “For the CONTENT of this test, please indicate your mental effort on the scale from 1 (very little) to 9 (very much);” the computer-based test cognitive load asked participants “For taking this test ON COMPUTER, please indicate your mental effort on the scale from 1 (very little) to 9 (very much).” A higher cognitive load indicates a greater mental effort.

### Procedure

After researchers explained the purpose of this study, voluntary participants signed a written informed consent and completed the surveys in a traditional classroom, which took about 30 min. One week later, each participant used a computer to take the SLW examination through a computer-based test in an Internet-connected computer classroom. At the end, participants recorded their perceived content and computer cognitive load for this examination. Most participants completed the examination within 30 min although they were allowed to take as much time as needed.

## Results

Table 1 presents the Pearson’s correlations with means and standard deviations of the variables observed in this study. Results showed that SLW anxiety had a significant negative correlation with SLW achievement. SLW anxiety had significant positive correlations with sleep disturbance, social inhibition and negative affective personality, as well as content and computer cognitive load. Furthermore, social inhibition personality had significant positive correlations with sleep disturbance, negative affectivity personality, as well as content and computer cognitive load. Content cognitive load had significant correlations with sleep disturbance and computer cognitive load.

**Table 1:** Means and correlations.

		Pearson correlation					
	Mean (SD)	1	2	3	4	5	6
1. Sleep disturbance	0.92 (0.62)	1					
2. Social inhibition personality	12.17 (4.51)	0.16^*^	1				
3. Negative affectivity personality	12.62 (5.18)	0.14	0.37^**^	1			
4. Content cognitive load	4.95 (1.69)	0.22^**^	0.26^**^	−0.05	1		
5. Computer cognitive load	4.40 (1.88)	0.10	0.18^*^	−0.07	0.69^**^	1	
6. SLW anxiety	68.92 (12.44)	0.23^**^	0.37^**^	0.22^**^	0.23^**^	0.23^**^	1
7. SLW achievement	8.31 (2.18)	−0.01	0.01	0.11	−0.15^*^	−0.20^**^	−0.20^**^

*N* = 172. SLW: Second language writing.

* *p* < 0.05;

** *p* < 0.01.

The results of t-tests showed no sex differences in any psychophysiological variables; except that women scored significantly higher than men on SLW achievement (mean difference = 5.22, *t* = −2.45, *df* = 171, *p* = 0.02). The purpose of this study is to investigate whether psychophysiological factors may predict SLW anxiety and achievement. Due to there being no sex differences on psychophysiological variables and a non-equivalent sex-ratio (women, *n* = 127; men, *n* = 45), we did not include sex in our SEM analyzes.

### SLW anxiety model

**Figure 1:**
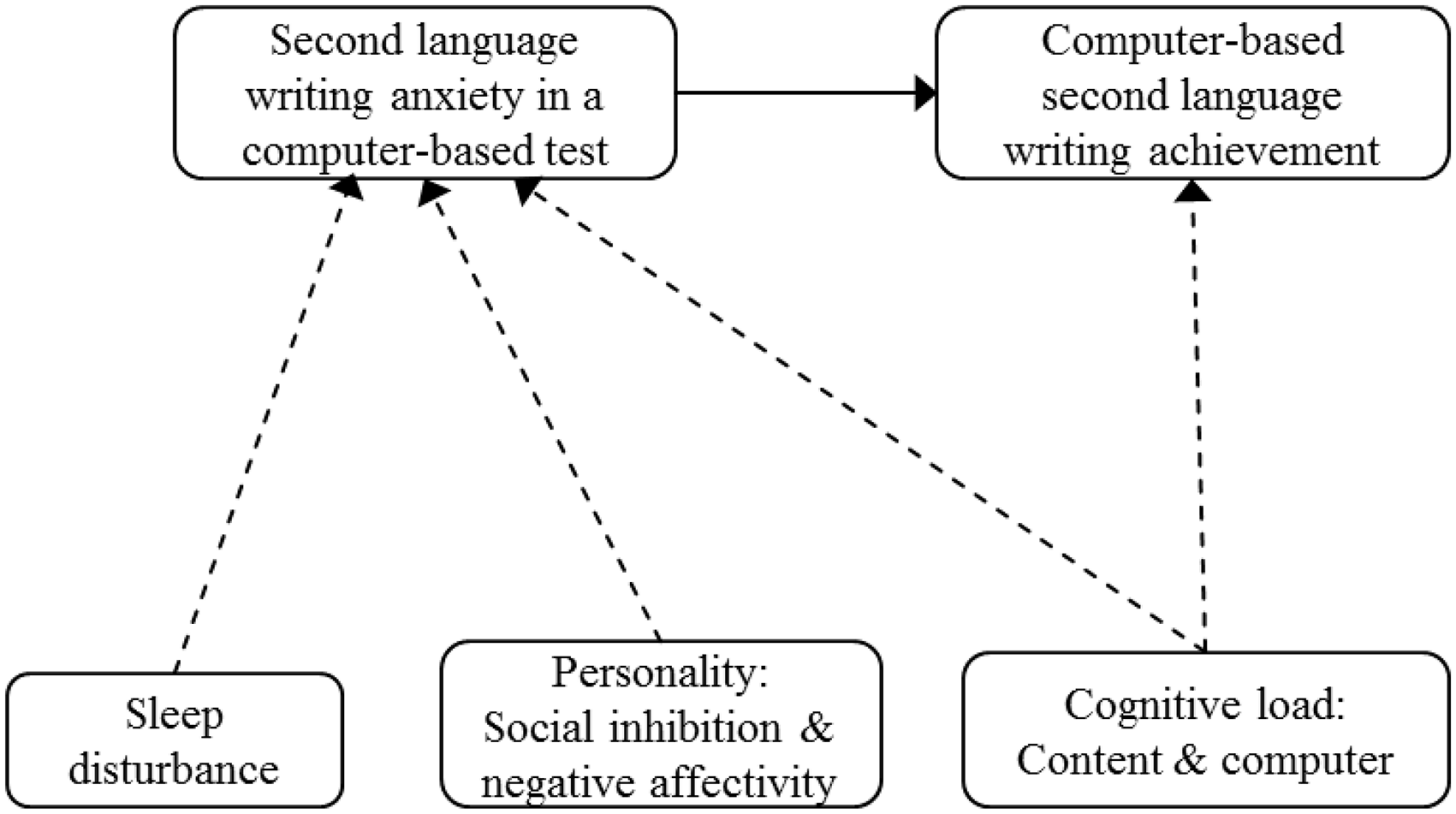
Proposed model. *N* = 172. Solid line: relation based on literature; Soft line: hypothesized relation.

We proposed a model (Figure 1) based on the significant correlations mentioned above and included paths: (a) from SLW anxiety in a computer-based test to computer-based SLW achievement, (b) from sleep disturbance, social inhibition and negative affectivity personality, and content and computer cognitive load to SLW anxiety in a computer-based test, and (c) from content and computer cognitive load on computer-based SLW achievement based on their significant correlations.

We performed maximum likelihood SEM analysis to examine the proposed model for an adequate-fit model. The sample size of this study (*n* = 172) is considered suitable for SEM analysis, as it exceeds 80, the lower bound for the adequate sample size for an eight-variable-model or 10 participants for each variable (Nunnally, 1967; Thompson, 2000). In addition, a sample size smaller than 100 or larger than 200 may result in a misguided model fit (Martens and Haase, 2006), which is not the case in this study.

**Figure 2:**
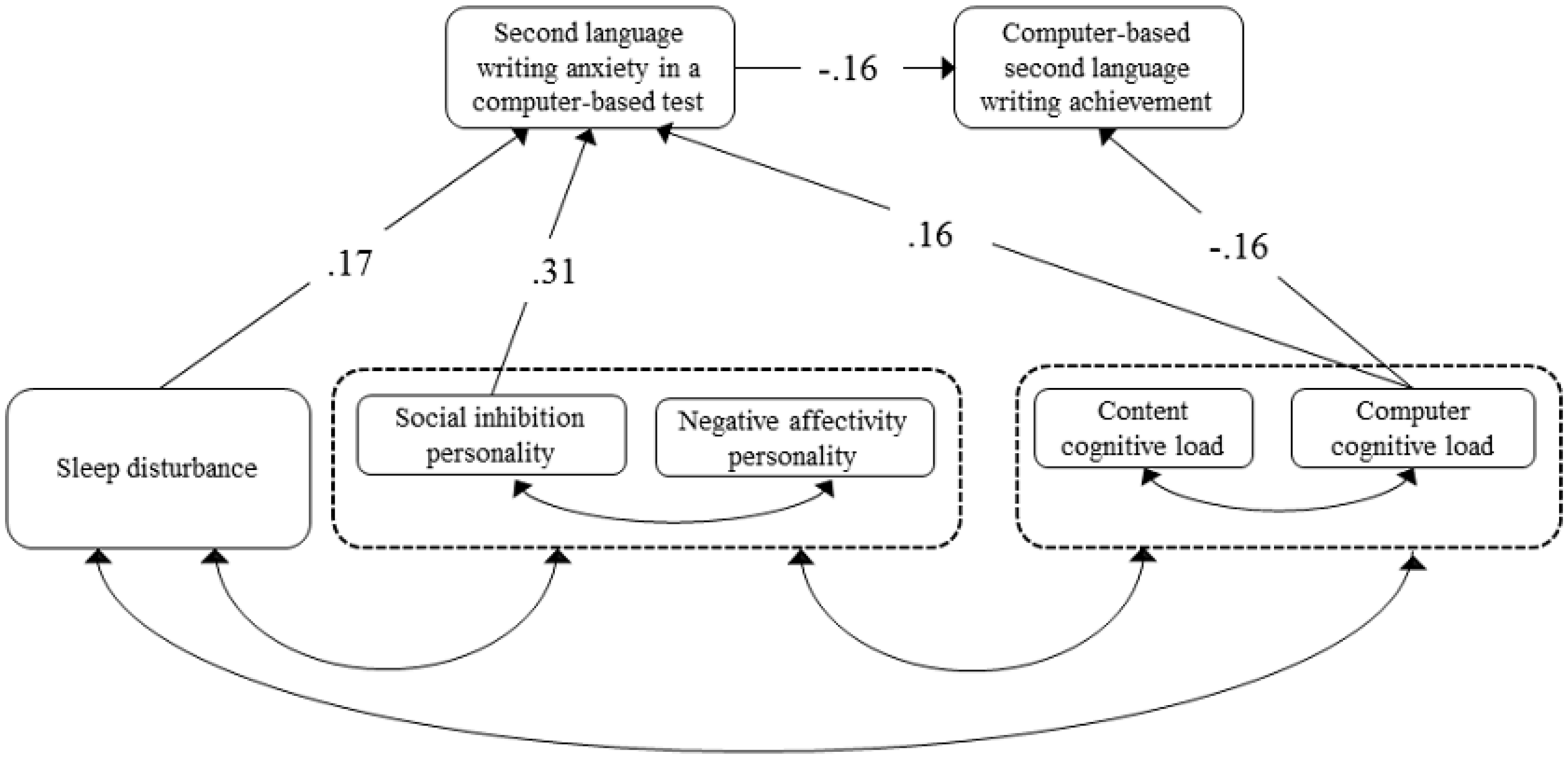
SLW Anxiety Model in A Computer-Based Test: Standardized regression coefficients and covariance. *N* = 172. Single-headed line: Significant standardized regression coefficients; Double-headed line: Covariance. Model *X*^2^ = 6.37, *df* = 6, *p* = 0.383, NFI = 0.97, CFI = 1.00, RMSEA = 0.02; R-squared multiple correlations: SLW anxiety a computer-based test = 0.19, computer-based SLW achievement = 0.07.

The proposed model was assessed based on the criteria of an adequate-fit model, including a non-significant value of p, a NFI or CFI larger than 0.95, and a RMSEA smaller than 0.05 (Hardy and Bryman, 2004; Loehlin, 2004). The results showed an adequate-fit SLW Anxiety Model in a computer-based test (*X^2^* = 6.37, *df* = 6, *p* = 0.383, *NFI* = 0.97, *CFI* = 1.00, *RMSEA* = 0.02) adjusted with covariance among psychophysiological factors (Figure 2). The R-squared multiple correlation of each endogenous variable, equivalent to the R-square in a regression equation, was 0.19 for SLW anxiety in a computer-based test and 0.07 for computer-based SLW achievement. Results indicate that having a social inhibition personality (the strongest predictor), sleep disturbance, and negative affectivity personality significantly increased SLW anxiety in a computer-based test. However, SLW anxiety in a computer-based test and computer cognitive load significantly decreased computer-based SLW achievement. Figure 2 illustrates the SLW anxiety model in a computer-based test with significant standardized regression coefficients of paths, and Table 2 presents the direct, indirect, and total effects of the paths.

**Table 2:** Standardized direct, indirect, and total regression coefficients in SLW anxiety model.

To	SLW anxiet			SLW achievement		
From	Direct	Indirect	Total	Direct	Indirect	Total
Sleep disturbance	0.17	---	0.17	---	−0.03	−0.03
Social inhibition personality	0.31	---	0.31	---	−0.05	−0.05
Computer cognitive load	0.16	---	0.16	−0.16	−0.03	−0.20
SLW anxiety	---	---	---	−0.16	---	−0.16

*N =* 172. ---: no coefficients.

## Discussion

Results showed several significant correlations. SLW anxiety in a computer-based test had a significant negative correlation with computer-based SLW achievement, supporting previous findings of the negative correlation between SLW anxiety and achievement in paper-and-pencil tests (Lee and Krashen, 1997; Woodrow, 2011; Park and French, 2013). Our findings also support the general view of the relations between anxiety and psychophysiological factors, in that SLW anxiety in a computer-based test had significant positive correlations with sleep disturbance (Killgore et al., 2008; Lund et al., 2010; Kim et al., 2011), social inhibition personality (Gest, 1997), negative affective personality (Denollet, 1991), as well as content and computer cognitive load (Paas et al., 2004; Sweller, 2004, 2010; Deleeuw and Mayer, 2008). Social inhibition and negative affectivity personality had a significant positive correlation, consistent with previous findings (Kret et al., 2011). Sleep disturbance had a significant positive correlation with social inhibition personality, supporting the view that sleep quality is correlated with intrapersonal and interpersonal functioning (Killgore et al., 2008).

The SLW Anxiety Model showed that SLW anxiety in a computer-based test and computer cognitive load had a negative association with computer-based SLW achievement. This finding indicates that the negative relation between SLW anxiety and SLW achievement was found not only in paper-and-pencil tests (Lee and Krashen, 1997; Woodrow, 2011; Park and French, 2013), but also in the computer-based test.

The SLW Anxiety Model showed that sleep disturbance, social inhibition personality, and computer cognitive load had positive associations with SLW anxiety in a computer-based test. Sleep disturbance was positively associated with SLW anxiety in a computer-based test, which is consistent with previous findings (Killgore et al., 2008; Lund et al., 2010; Kim et al., 2011). From the analysis of SEM structure, sleep disturbance is directly positively associated with SLW anxiety but is not directly negatively associated with SLW achievement, which might indirectly negatively impact computer-based SLW achievement *via* higher SLW anxiety.

Social inhibition personality was the strongest predictor of second language computer-based writing anxiety; this finding appeared to be supported from the view that people having a higher social inhibition personality tend to feel more stressed than others (Gest, 1997), and underscores the importance of reducing such stressful feelings in a learning environment. Social inhibition personality not only negatively impact communicative foreign language performance (Patterson and Ritts, 1997; King and Smith, 2017) but also negatively impact second language writing anxiety.

Computer cognitive load was positively associated with SLW anxiety in a computer-based test in the current study, which is consistent with previous findings (Paas et al., 2004; Sweller, 2004, 2010; Deleeuw and Mayer, 2008). From the analysis of SEM structure, computer cognitive load is positively associated with SLW anxiety whereas is negatively associated with SLW achievement. Our findings indicate that computer cognitive load, a unique factor in computer-based tests, carried the same weight as computer-based SLW anxiety in predicting SLW achievement in a computer-based test. These outcomes suggest that decreasing computer cognitive load is as crucial as decreasing SLW anxiety in order to enhance SLW achievement in a computer-based test. Our findings suggest that sleep disturbance, social inhibition, and computer cognitive load are the crucial factors to be considered in reducing SLW anxiety in computer-based tests.

Several pedagogical strategies may modify psychophysiological factors and reduce the SLW anxiety in computer-based tests. For example, for students who are considered having a social inhibition personality, instruction may include anxiety coping skills (Davis et al., 2000) or guide them to engage in conversations with English-speaking people to ease their fear of social interaction. Also, instructions for computer usage should provide more practice and guidance in the use of computers for writing, thus reducing the computer cognitive load. In a broader context, instructors may encourage or train students to adopt effective learning strategies. For instance, in learning a foreign language, students could be instructed to practice retrieving content from memory rather than re-reading text or reviewing material, since recalling makes the learning stronger and more easily recalled again later (Brown et al., 2014). Students should be advised how to achieve sufficient sleep, since sleep helps to encode and consolidate newly learned information, which may make the retrieval of learned information quicker and easier, and decrease the content cognitive load in learning a foreign language.

### Limitations and future research

Some limitations should be acknowledged in interpreting the results of this study. (a) We are aware that the SLW Anxiety Model in a computer-based test may not be the only model, and other models may also adequately explain the relations among psychophysiological factors and SLW anxiety and achievement in a computer-based test. In addition, the SLW Anxiety Model in a computer-based test had low R-squared multiple correlations (0.19 for SLW anxiety and 0.07 for SLW achievement), or a low variance accounted for by the model. In future studies, incorporating more influential factors into the SLW Anxiety Model in a computer-based test may improve the explanation of the model. (b) All participants were English majors from one university, which limits their representativeness. Replication of this study with a larger and more diverse sample is recommended. (c) The data of this study were collected through self-report measures, of which the responses may be adjusted to meet social expectations and result in bias (Nisbett and Wilson, 1977); and (d) Content and computer cognitive load were each assessed by one-item measure. We feel confident in its face validity since it is a commonly used mental-effort rating scale in previous studies (Brunken et al., 2003; Deleeuw and Mayer, 2008). However, we do acknowledge that multi-item measures would be preferable. Further research on developing multi-item measures for cognitive load is suggested.

## Data availability statement

The raw data supporting the conclusions of this article will be made available by the authors, without undue reservation.

## Ethics statement

The studies involving human participants were reviewed and approved by Asia University Medical Research Ethics Committee. The participants provided their written informed consent to participate in this study.

## Author contributions

S-PL, SC, and S-DL contributed to the conceptualization. S-PL, SC, H-KS, and Z-YC contributed the methodology. S-PL drafted the manuscript. S-PL, H-KS, SC, Z-YC, and S-DL edited and revised the manuscript. All authors approved the final version of the manuscript.

## Funding

The study was supported by grant CMU98-ASIA-07 from Asia University and China Medical University. This study was partially supported by China Medical University and Weifang Medical University for data validation. The funders were not associated with design, data searching, data collection, synthesis, and publication decisions.

## Conflict of interest

The authors declare that the research was conducted in the absence of any commercial or financial relationships that could be construed as a potential conflict of interest.

## Publisher’s note

All claims expressed in this article are solely those of the authors and do not necessarily represent those of their affiliated organizations, or those of the publisher, the editors and the reviewers. Any product that may be evaluated in this article, or claim that may be made by its manufacturer, is not guaranteed or endorsed by the publisher.
